# Integration of the Integrated Emergency Surgical Officers (IESO) initiative within the health system: Perspectives of non-physician surgeons in Ethiopia’s Tigray region

**DOI:** 10.1371/journal.pone.0358569

**Published:** 2026-09-28

**Authors:** Hale Teka, Mussie Alemayehu, Mengistu Hagazi Tequare, Hailesllassie Berhe, Hailemariam Gebrearegay Haileeyesus, Rahel Nardos

**Affiliations:** 1 Department of Obstetrics and Gynecology, Faculty of Medicine, College of Health Sciences, Mekelle University, Mekelle, Ethiopia; 2 Department of Reproductive Health, Faculty of Public Health, College of Health Sciences, Mekelle University, Mekelle, Ethiopia; 3 Department of Health Systems, Faculty of Public Health, College of Health Sciences, Mekelle University, Mekelle, Ethiopia; 4 Department of Anesthesiology, Critical Care, and Pain Medicine, Faculty of Medicine, College of Health Sciences, Mekelle University, Mekelle, Ethiopia; 5 Department of Pediatrics and Child Health, Faculty of Medicine, College of Health Sciences, Mekelle University, Mekelle, Ethiopia; 6 Department of Obstetrics, Gynecology, and Women’s Health, University of Minnesota, Minneapolis, Minnesota, United States of America; Menzies School of Health Research: Charles Darwin University, AUSTRALIA

## Abstract

**Background:**

Task-shifting and task-sharing are strategies for redistributing or collaboratively delivering clinical tasks within health workforce teams to address shortages and expand access. In Ethiopia, Integrated Emergency Surgical Officers (IESOs) were introduced to provide emergency obstetric and essential surgical care in underserved areas. In Tigray, conflict-related damage, displacement, workforce attrition, and disruption of referral and training systems intensified pre-existing surgical workforce shortages. Evidence on how IESOs have been integrated, supported, and retained in this fragile health-system context remains limited. This study assessed the status and experiences of IESOs in Tigray and identified strategies for strengthening their integration and retention.

**Methods:**

We conducted a concurrent mixed-methods study in November 2024. Of the 84 IESOs practising in Tigray, 59 attended the region-wide safe-cesarean-delivery training and all 59 completed the survey, giving a 100% response rate among invtied participants and representing 70.2% of practising IESOs in the region. Two focus group discussions with nine participants each and five in-depth interviews were conducted. Quantitative data were analyzed descriptively using R version 4.2.2, and qualitative data were analyzed thematically. Findings were integrated through triangulation and a joint display.

**Results:**

Participants worked across general hospitals, primary hospitals, and health centers. Most strongly agreed that IESOs contributed to reducing maternal mortality, 54(91.5%) and perinatal or neonatal mortality 48 (88.1%). The proportions who were very likely to remain in the profession declined from 31 (52.5%) at three years to 24 (40.7%) at five years and 7 (11.9%) at ten years, demonstrating a pronounced projected retention cliff. Resource shortages, limited managerial recognition, inter-professional conflict, and absent career-progression pathways were the dominant challenges, whereas community acceptance and the opportunity to initiate lifesaving services in remote settings were major sources of motivation.

**Conclusion:**

IESOs extend emergency obstetric and surgical services to underserved communities in Tigray, but their long-term retention is precarious. A sustainable program requires a nationally and regionally defined scope of practice, an appropriate salary and professional grading structure, funded continuing professional development, formal IESO specialization or advanced clinical career pathways, reliable surgical supplies and infrastructure, and structured collaborative governance with surgeons and obstetrician-gynecologists.

## Background

The global shortage and unequal distribution of skilled health workers remain major barriers to universal health coverage [1,2]. The World Health Organization distinguishes task-shifting, in which selected tasks are transferred to appropriately trained cadres, from task-sharing, in which responsibility is delivered collaboratively across a team according to competence, regulation, and supervision [3,4]. Both approaches can expand coverage, shorten delays, and improve continuity of essential services when accompanied by adequate training, governance, referral systems, and resources [5,6]. Mid-level health professionals can also be trained more rapidly and at lower cost than physicians and may be more likely to remain in rural areas [7].

Historically, task-shifting has been leveraged by various countries in response to crises or persistent health workforce shortages. During the French Revolution (1789–1794), for instance, the collapse of formal medical education led to the recruitment of young men with limited training as health officers, or *officiers de santé*, after they had acquired a set period of hands-on clinical instruction [8]. In Russia, “Feldshers”—trained as physician assistants—were deployed from the mid-19th century onward to serve remote communities, and their role continued beyond the Bolshevik Revolution in 1917 [9]. In China, following the Cultural Revolution in 1965, “barefoot doctors” became a cornerstone of rural healthcare, focusing on preventive and basic curative services to reach underdeveloped regions [10].

In many low- and middle-income countries (LMICs), community health workers (CHWs) have likewise been central to task-shifting initiatives for decades. CHWs—often lay individuals from the community—are trained to provide basic interventions, health education, and referrals, demonstrating considerable success in improving maternal and child health as well as managing common infectious diseases [11–13]. Their widespread adoption underscores the feasibility of transferring select tasks from higher-level professionals to trained lay or mid-level workers [6,14].

Within sub-Saharan Africa, numerous countries rely heavily on nonphysician clinicians (NPCs)—health workers trained to undertake many diagnostic and clinical functions typically performed by physicians [14]. Mullan et al. found that in 25 out of 47 countries surveyed, NPCs complement or outnumber physicians [15]. Notably, all these 25 countries fall below the 2.3 skilled health workers (physicians, nurses, midwives) per 1000 population threshold deemed essential for adequate coverage of key interventions such as skilled birth attendance [16]. Deploying NPCs has been associated with better access to HIV/AIDS care, obstetric services (e.g., cesarean sections), and essential surgical procedures [17,18].

Ethiopia, the second most populous country in Africa, has similarly grappled with shortages and maldistribution of skilled health professionals—particularly in rural regions [19,20]. In response, the Federal Ministry of Health (FMoH) introduced multiple initiatives. One major effort was the Health Extension Program (HEP), which trains and deploys CHWs to deliver preventive and primary healthcare interventions at the community level [21]. Beyond community cadres, the FMoH accelerated the training of mid-level providers through specialized programs such as Integrated Emergency Surgical Officers (IESOs), Clinical Midwifery, Master’s in Infectious Disease, and Master’s in Maternal and Child Health [22]. IESOs, for instance, are often BSc-level nurses, midwives, or health officers who undergo a three-year master’s program to perform critical obstetric and general surgical interventions—including cesarean sections, laparotomies, and other emergency procedures [23]. A study outside Tigray suggest that IESOs have been instrumental in reducing maternal and newborn mortality and have lowered referrals to higher-level facilities [24,25].

However, the role and integration of IESOs in Tigray remain understudied, particularly after the recent conflict. Damage to health facilities, displacement of professionals, disrupted referral pathways, and interrupted training and supply systems compounded the region’s pre-existing shortage and maldistribution of surgical personnel [26]. Although IESOs reportedly continued providing emergency obstetric and surgical care during and after the crisis, empirical evidence on their working conditions, professional relationships, retention intentions, and career expectations is scarce. This study therefore examined IESOs’ perceptions of their training, professional integration, institutional support, and future careers to inform health authorities, academic institutions, and professional bodies responsible for sustaining the cadre.

## Methods and materials

### Study setting, design, and period

This research was conducted in Tigray Regional State, Ethiopia, in November 2024. The regional health system follows Ethiopia’s three-tier structure, comprising primary, secondary, and tertiary facilities. Conflict-related damage, insecurity, workforce displacement, and shortages of medicines, equipment, blood, and power continued to disrupt access and service delivery in many areas during the study period [26].

We used a concurrent mixed-methods design. The quantitative component was a cross-sectional survey of IESOs attending three short training cohorts in Mekelle. The qualitative component comprised focus group discussions (FGDs) and in-depth interviews (IDIs). Both components were collected during the same period, analyzed separately, and integrated at interpretation through convergence, complementarity, and dissonance, including a joint display linking numerical findings with explanatory themes.

### Study population and sampling

At the time of the study, 84 IESOs were practising in Tigray [27,28]. The region-wide safe-cesarean-delivery training was organized in Mekelle in three consecutive cohorts during November 2024. Fifty-nine of the 84 practising IESOs attended the training. We used consecutive sampling and invited all 59 attendees to participate in the survey; all consented and completed the questionnaire, yielding a 100% response rate among training attendees and coverage of 70.2% of the practising IESO workforce in Tigray. The remaining 25 practising IESOs did not attend the training and were therefore not approached for the survey. For the qualitative component, participants were purposively selected to capture variation in age, professional experience, geographic location, and facility type. Two FGDs, each involving nine participants, and five IDIs were conducted.

### Data collection tools and procedures

Three structured instruments were developed for the broader training and study activities (S1 File). The 87-item study questionnaire was informed by a literature review [3,5,13,25] and multidisciplinary expert consultation and covered sociodemographic characteristics, perceived training adequacy, self-reported competence, job satisfaction, institutional support, professional relationships, and career intentions. The instrument was reviewed for face and content relevance but was not subjected to formal psychometric validation; this is acknowledged as a limitation. A separate pre-test/post-test assessed training-related knowledge and skills, and a course-evaluation form assessed participants’ experience of the training. Only variables relevant to the present study are reported. Two instruments were administered through KoboToolbox on participants’ smartphones; the knowledge assessment used paper forms. MA and MH guided participants how to download and install KoboToolbox, how to respond, and how to submit data after attempting all set of questions.

Qualitative data were collected using semi-structured FGD and IDI guides exploring motivation, professional identity, experiences before, during, and after the conflict, relationships with other professionals, institutional support, resource constraints, and future aspirations. Experienced researchers facilitated the sessions in private settings. Discussions and interviews were audio-recorded with permission, transcribed verbatim, and translated from Tigrinya into English. Sampling across primary hospitals, general hospitals, and health centers was sought; however, because participation depended on training attendance, exact proportional balance by facility level was not imposed.

### Data analysis

Quantitative data were exported from KoboToolbox to R version 4.2.2 for cleaning and analysis. Categorical variables are reported as frequencies and percentages; continuous variables, where applicable, are summarized using means and standard deviations. Likert-type items were analyzed as individual ordered categorical items rather than combined into an unvalidated composite scale. Accordingly, internal-consistency statistics were not calculated. The three-, five-, and ten-years retention questions were separate prospective items asking how likely respondents were to continue working as IESOs at each time horizon. Because the study was exploratory, the sample was small, and participant-level data available for this revision did not support a prespecified adequately powered multivariable model, no inferential predictor analysis was undertaken; findings are interpreted descriptively.

Qualitative data were analyzed manually using a reflexive thematic-analysis approach. Researchers first familiarized themselves with the transcripts, generated inductive codes, and iteratively organized related codes into candidate themes. Two researchers coded the material independently during the initial phase and compared interpretations; disagreements were discussed to refine the coding framework rather than quantified as inter-coder reliability. Themes were reviewed against the coded extracts and full dataset, defined, and named. Integration occurred after separate analyses by comparing areas of convergence, complementarity, and divergence and by constructing a joint display that links quantitative indicators, qualitative explanations, and health-system implications.

### Ethical considerations

The Institutional Review Board of the College of Health Sciences, Mekelle University approved the study (MU-IRB 2345/2024), including the use and documentation of written informed consent. Before participation, the research team tasked at data collection (MA and MH) explained the study, voluntary participation, confidentiality, and the right to withdraw. Consent was recorded through a mandatory confirmation field before the electronic questionnaire could be opened and documented by the moderator or interviewer before each recorded FGD or IDI; the data collector served as witness to the consent process. No names were collected. Potentially identifying characteristics were minimized by reporting age in broad categories and omitting sex from quotations. Data were anonymized and handled confidentially.

## Results

### Quantitative findings

#### Sociodemographic characteristics.

A total of 59 IESOs participated. All 59 IESOs who attended the region-wide training were approached and completed the survey, corresponding to a 100% response rate among training attendees. Nearly three-fourths, 44 (74.6%) were aged 40 years or younger, while 15 (25.4%) were above 40 years. The majority were married, 47 (79.7%), followed by single, 8 (13.6%) and divorced, 4 (6.8%). Most respondents, 52 (88.1%) had previously been health officers before joining the IESO program, and over 70% had eight or more years of work experience (Table 1).

**Table 1:** Sociodemographic characteristics of IESOs in Tigray, Ethiopia, 2024 (n = 59).

Variable	Categories	Number (%)	n (%) [95% CI]
Age (years)	≤ 40	44 (74.6)	44 (74.6) [62.2–83.9]
	> 40	15 (25.4)	15 (25.4) [16.1–37.8]
Marital Status	Married	47 (79.7)	47 (79.7) [67.7–88.0]
	Divorced	4 (6.8)	4 (6.8) [2.7–16.2]
	Single	8 (13.6)	8 (13.6) [7.0–24.5]
Type of Health Facility	General Hospital	20 (33.9)	20 (33.9) [23.1–46.6]
	Primary Hospital	19 (32.2)	19 (32.2) [21.7–44.9]
	Health Center	20 (33.9)	20 (33.9) [23.1–46.6]
Profession before IESO	Health Officer	52 (88.1)	52 (88.1) [77.5–94.1]
	Nurse	7 (11.9)	7 (11.9) [5.9–22.5]
Work Experience (years)	≤ 7	16 (27.2)	16 (27.1) [17.4–39.6]
	≥ 8	43 (72.8)	43 (72.9) [60.4–82.6]

#### Perceived impact on maternal and neonatal health.

When asked about the impact of the IESO program on maternal and newborn health, 54 (91.5%) “strongly agreed” that it has contributed to reducing maternal mortality, and 52 (88.1%) “strongly agreed” that it has reduced perinatal and neonatal mortality. Furthermore, half of the respondents, 30 (50.8%) strongly recommended the IESO profession to others. A total of 51 (86%) respondent strongly believed that having IESOs in rural areas improves healthcare access for underserved communities.

#### Collaboration, recognition, and support.

Although 33 (55.9%) respondents rated collaboration with other healthcare professionals as very good, only 9 (15.3%) rated collaboration with management bodies as very good, and 6 (10.2%) rated administrative support as very good. Twenty-eight respondents (47.5%) agreed that management adequately recognized their work; conversely, 31 (52.5%) did not select the ‘agree’ category, indicating substantial perceived deficits in managerial recognition. Community appreciation was considerably higher: 48 (81.4%) agreed that local residents adequately appreciated their work.

#### Conflict of interest and acceptance by specialists.

Conflicts with surgeons and obstetrician-gynecologist (OB/GYN) specialists were reported at varying intensities: 7 (11.9%) indicated a “very high” level of conflict with surgeons, and 6 (10.2%) reported a “very high” conflict with OB/GYN specialists. Acceptance by surgeons was rated as “very low” by 6 (10.2%) of participants. Some participants cited competition in private clinics and unclear scopes of practice as sources of friction.

#### Likelihood of continuing as IESOs.

The proportion reporting that they were very likely to continue as IESOs declined from 51 (52.5%) at three years to 24 (40.7%) at five years and 7 (11.9%) at ten years. These separate time-horizon items show a projected retention cliff rather than a validated overall retention scale (Table 2 and Fig 1).

**Table 2:** Selected indicators on program impact, collaboration, and future outlook (n = 59).

Indicator	Category	Number (%)
Strongly recommend IESO profession	Yes	30 (50.8)
IESO contribution to ↓ maternal mortality	Strongly agree	54 (91.5)
IESO contribution to ↓ neonatal mortality	Strongly agree	52 (88.1)
Collaboration with management body	Very good	9 (15.3)
Support from hospital/district administration	Very good	6 (10.2)
Adequate community appreciation	Agree	48 (81.4)
Adequate recognition by management	Agree	28 (47.5)
Very high conflict of interest with surgeons	Very high	7 (11.9)
Very low acceptance by surgeons	Very low	6 (10.2)
Very likely to continue (3 years)	Very likely	31 (52.5)
Very likely to continue (10 years)	Very likely	7 (11.9)

**Fig 1:**
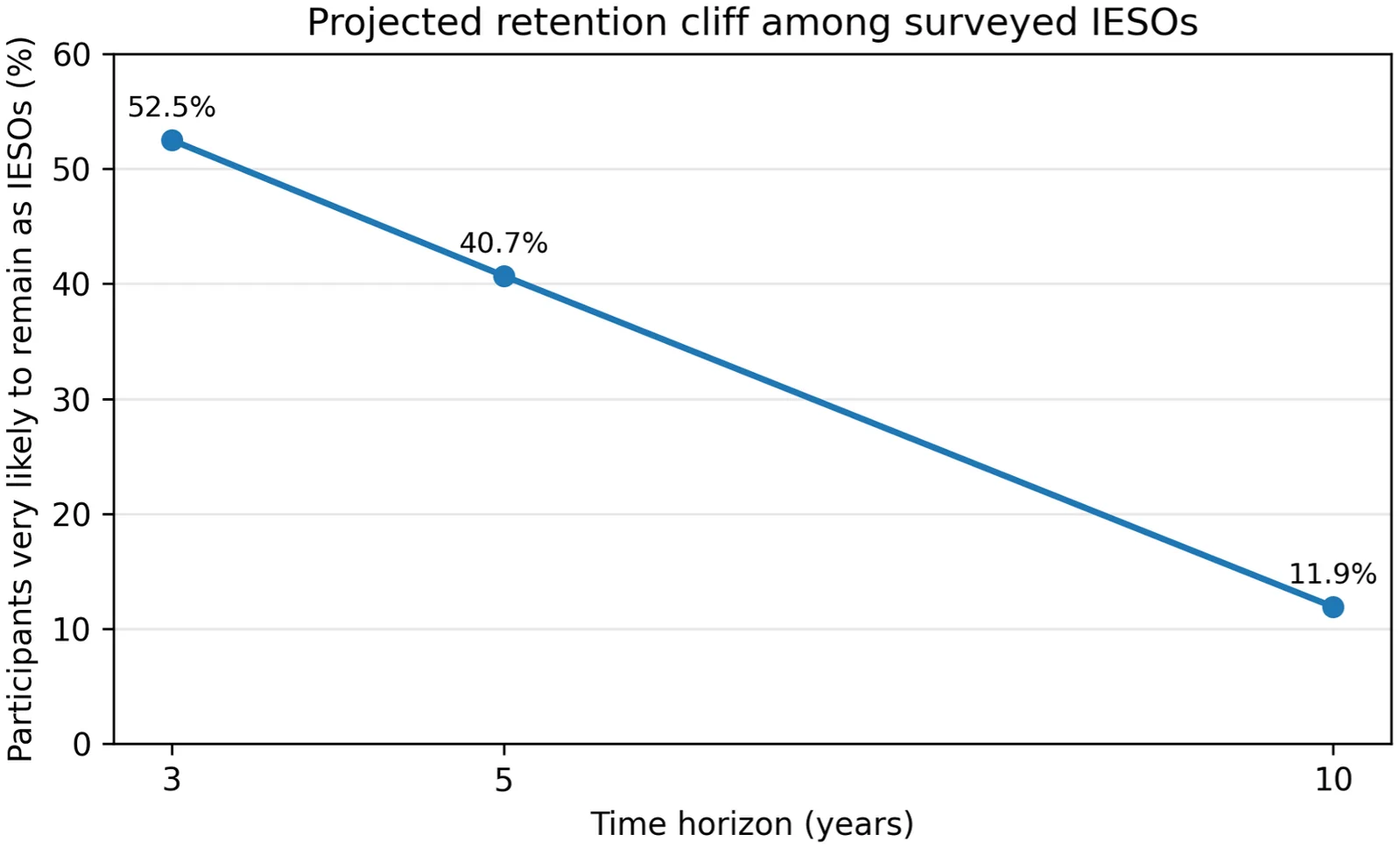
Projected retention cliff among surveyed IESOs (n = 59). The percentage self-reporting that they were very likely to remain as IESOs at three, five, and ten years is shown, collected at a single time point.

#### Qualitative findings.

The two FGDs (nine participants each) and five IDIs generated three overarching themes: (1) health-system and professional-integration challenges; (2) perceived contribution to service initiation and mortality reduction; and (3) career development and future aspirations. The qualitative findings explained why favorable perceptions of community impact coexisted with weak long-term retention intentions. Table 3 presents the mixed-methods joint display.

**Table 3:** Mixed-methods joint display integrating quantitative and qualitative findings.

Quantitative finding	Qualitative explanation	Integrated interpretation	Health-system implication
Very likely to remain: 52.5% at 3 years, 40.7% at 5 years, 11.9% at 10 years	No career path, limited incentives, and plans to leave	A projected retention cliff develops despite commitment to service	Create salary, grading, promotion, continuing education, and specialization pathways
Management collaboration rated very good by 15.3%; administrative support by 10.2%	Participants described limited representation and institutional recognition	Weak organizational integration undermines professional identity and morale	Formal representation, supervision, appraisal, and joint governance mechanisms
81.4% agreed that communities adequately appreciated their work	Community trust and visible lifesaving impact motivated continued rural service	Community legitimacy is a major retention asset but cannot compensate for structural deficits	Preserve rural deployment while improving working conditions and recognition
91.5% and 88.1% strongly perceived reductions in maternal and neonatal/perinatal mortality, respectively	Narratives described initiation of surgical services and avoided referrals	Perceived impact is plausible and strongly held, but remains self-reported	Link IESO deployment to routine registry, referral, safety, and outcome monitoring

#### Challenges within the health system.

Almost all participants described significant resource shortages that hindered continuous and high-quality service provision. They frequently mentioned a lack of essential drugs, power backups, and blood transfusion services. One discussant stated:

“I could not serve what I should due to lack of equipment and medicine. I have seen lives that could be saved been lost.” (>40-year-old, Married, FGD)

Another participant echoed the same sentiment:

“Even currently in the area I have been working, I do not believe as I am giving enough service that is supposed to be given as my profession. There is resource shortage, lack of manpower, lack of power backup, lack of blood transfusion and other different challenges. As a result, there is interruption of services and cases are referred. I believe, I am not providing service that is expected from me.” (≤40-year-old, Married, FGD)

IESOs also expressed frustration with limited support and recognition from higher-level leadership:

“Nobody stands for us in Tigray regional health bureau as well [as the] minister of health. A lot of attempts were tried to request our rights, but no one could answer them. We are not in any merit. We are not in [a] professional category.” (>40-year-old, Married, FGD)

Beyond systemic resource gaps, conflicts of interest with surgeons and sometimes OB/GYN specialists were common. Participants believed some surgeons viewed them as competitors for limited surgical cases or private-sector income. This tension was vividly described by one participant:

“We are victims. We do not have recognition. Our association is not recognized. The only thing needed from us is our work… Especially when it comes to [the] private clinic market. Defaming and other things are seen. It goes to [the] regional health bureau. I know [an] IESO [who has] been fired from his work due to such things.” (>40-year-old, Married, FGD)

Such disputes often centered on professional identity and perceived encroachment on surgeons’ traditional responsibilities:

“They [surgeons] used to say ‘Those are our future bread takers.’ They used to look at us as their enemy. They used to fear we will be taking their market.” (>40-year-old, Married, FGD)

In some cases, the conflict had escalated to the extent that IESOs contemplated leaving:

“We do not have any need to stay. We all are searching [for] means to go out. Generally, we are in such a condition.” (>40-year-old, Married, FGD)

#### Perceived impact on service initiation and mortality reduction.

Despite these systemic challenges, IESOs emphasized their significant contributions to improving maternal and child health outcomes in remote facilities. Many described the satisfaction of initiating surgical services where none had existed before, thus reducing referrals to distant hospitals. One participant illustrated this sense of accomplishment:

“In the beginning, since the health centers were not providing surgical services, especially in the first two to three cases, I was begging or lobbying mothers to receive the service. Later after they observe the good outcomes, the community provides us acceptance and recognition.” (>40-year-old, Married, IDI)

Participants narrated dramatic life-saving interventions performed under extremely constrained conditions. One of the participants recounted a complicated case involving both cesarean section and bowel surgery performed overnight without the possibility of consulting a specialist:

“A mother came… referred from a health center at midnight. I found gangrene of the bowel and ileum. I was with an assistant nurse and anesthetist. I could not consult anyone. I did a colostomy and ileo-transverse anastomosis alone. The mother survived… The child is now 6 or 7 years old… This mother could show the significance of this profession very well.” (>40-year-old, Married, FGD)

Such testimonials reinforce the quantitative findings that IESOs believe their work substantially reduces maternal and neonatal mortality. Another participant summarized:

“We have served many mothers. We have contributed to the reduction of maternal and child deaths. It is a responsible profession serving in peripheries.” (≤40-year-old, Married, FGD)

Participants also underscored the strong support they receive from their local communities, who see firsthand how IESOs mitigate travel costs, referral delays, and other burdens:

“I got married and live in the area. I had opportunities to go to towns like Mekelle. The relationship created in the community does not give me a motive to go to other areas. The main reason we continue in this profession is the acceptance and recognition by the community.” (<40-year-old, Married, FGD)

#### Career development and future aspirations.

IESOs highlighted the lack of a defined career path as a critical barrier to staying in the profession long-term. Many pointed out that they had minimal opportunities for advanced clinical training or specialization:

“We are interested [in continuing to a] PhD academically in clinical [areas], not public health. We were requesting all possible options… but we have no career path. The program does not consider the vision of individual development of future careers.” (>40-year-old, Married, IDI; ≤40-year-old, Married, IDI)

Some lamented the apparent discontinuation or slowdown of the IESO training program in Tigray:

“Why did Tigray stop teaching? Do you think it is due to budget constraints? The justification they provide is there was a donation for educating IESO, and then the donation stopped.” (>40-year-old, Married, FGD)

Despite a lack of incentives and formal recognition, participants consistently affirmed their commitment to serving rural communities—largely driven by community gratitude and the moral imperative to save lives:

“We are traumatized with a lot of things more than anyone. We are serving more than anyone. Our duty is not incentivized… This [training] is my second time to attend since I am IESO… But I will keep serving society.” (>40-year-old, Married, FGD)

## Discussion

This mixed-methods study examined the integration and future of IESOs in a conflict-affected regional health system. Respondents perceived the cadre as central to expanding emergency obstetric and surgical access, yet their intention to remain fell sharply across longer time horizons. The projected retention cliff—from 52.5% at three years to 11.9% at ten years—appears to reflect accumulated structural pressures rather than dissatisfaction with the social value of the work. Quantitative evidence of weak managerial support and recognition converged with qualitative accounts of resource scarcity, professional marginalization, and absent career pathways.

Historical precedents,including the experiences of officiers de santé during the French Revolution, Feldshers in Russia, and barefoot doctors in China,demonstrate that mid-level cadres have repeatedly filled critical service gaps during and after crises [8–10]. Contemporary evidence from sub-Saharan Africa, including Mozambique’s physician assistant model for obstetric surgery and various task-shifting initiatives for HIV care, reinforces the notion that nonphysician clinicians can deliver high-quality services [4,17,29–32]. These gains often hinge on clear regulatory support, adequate training, and stable supplies of essential resources [1,3,6,14]. Analogous to findings in other settings, IESOs in Tigray continue to struggle with shortages of pharmaceuticals, operating room equipment, and reliable power sources, especially in health facilities still recovering from conflict-related disruptions [5,26].

Professional conflict was another important barrier. Participants described competition and uncertainty about professional boundaries, patterns also reported where mid-level surgical cadres and physicians have overlapping roles without explicit regulation [14,15,33]. These tensions may be intensified by gendered and hierarchical professional norms, although sex-disaggregated analysis was intentionally unavailable because identifying characteristics were minimized in this small professional community. Clear scopes of practice, joint clinical governance, referral and supervision arrangements, and inter-professional training are therefore necessary to shift the relationship from perceived substitution toward safe task-sharing.

Limited career progression similarly constrained motivation and retention. Participants wanted advanced clinical education, specialization, and formal recognition rather than being restricted to a static professional grade. International experience suggests that retention of mid-level cadres depends not only on intrinsic motivation but also on equitable salary scales, professional titles, promotion criteria, continuing education, and credible routes to advanced practice [10,15,21]. In Tigray, conflict-related disruption of training institutions, professional networks, and financing likely magnified these longstanding weaknesses.

Despite these systemic shortcomings, IESOs in this study reported strong community appreciation, deriving motivation from the trust and gratitude of local populations [34]. This finding is consistent with a recent life-history study of IESOs in Tigray, which documented strong community acceptance alongside severe supply shortages, excessive workload, professional conflict, limited government support, unclear career pathways, and scarce continuing professional development opportunities [27]. A subsequent mixed-methods evaluation involving the same regional training platform showed substantial improvement in safe-cesarean-delivery knowledge among 59 IESOs, while participants continued to emphasize the need for ongoing mentorship, career development, and system support [28]. Together, these findings suggest that intrinsic motivation and episodic training are valuable but insufficient without sustained institutional investment.

Lessons from task-shifting programs worldwide illustrate that key enablers include explicit scopes of practice, adequate and sustained resource provision, structured professional development, and collaborative relationships with fully trained specialists [1,6,14,33,35]. Strengthening regulatory frameworks and clearly defining the roles of IESOs may lessen professional conflicts and enhance teamwork. Ensuring stable supply chains and adequate infrastructure is critical if IESOs are to perform advanced procedures safely. Formalizing educational and career opportunities can help retain experienced IESOs and attract new practitioners, a pressing concern in conflict-affected regions. Collaborative engagement with existing physician teams, possibly through joint training, could also reduce tensions and reinforce a shared sense of mission.

Taken together, the findings support a concrete health-system response: define and legally endorse the IESO scope of practice; establish an appropriate salary, grading, and promotion structure; create funded continuing professional development and advanced clinical or IESO-specialization pathways; guarantee minimum surgical infrastructure, blood, power, medicines, and equipment; and institutionalize collaborative governance with surgeons, obstetrician-gynecologists, anesthesia professionals, and referral facilities. These measures are more specific than general calls for stronger regulation and are likely to be central to retention in fragile and resource-constrained settings.

The mixed-methods design is a strength because qualitative accounts helped explain the numerical patterns. Several limitations should nevertheless guide interpretation. First, although the survey included 59 of the 84 practising IESOs in Tigray (70.2%) and achieved a 100% response rate among invited participants, sampling was restricted to those who attended the region-wide training in Mekelle. IESOs unable to attend because of distance, insecurity, workload, or service constraints may have had different experiences, creating potential selection and sampling bias. Second, the modest sample size limited statistical power and the precision of subgroup comparisons. Third, administering the survey during a professional training program may have encouraged favorable responses about training needs or professional experiences. Fourth, the study relied on self-reported perceptions, including perceived contributions to mortality reduction, rather than objective registry, referral, complication, or mortality data. Recall, social desirability, and interviewer biases are therefore possible. Fifth, the cross-sectional assessment captures a snapshot from November 2024 and cannot establish causality or changes over time. Finally, the findings are specific to Tigray’s conflict-affected health system and may not be directly generalizable to other regions.

## Conclusion

IESOs are an important component of emergency obstetric and surgical service delivery in Tigray, particularly in underserved facilities. Their strong sense of community value coexists with a steep projected retention cliff driven by resource shortages, weak managerial recognition, professional conflict, and limited career progression. Sustaining the cadre requires a formally defined scope of practice, equitable salary and grading structures, funded continuing education, advanced clinical or specialization pathways, reliable surgical infrastructure and supplies, and collaborative governance with specialist teams. Because reported mortality benefits were based on professional perceptions, future evaluations should confirm impact using objective service and outcome data.

## Supporting information

S1 File Supplemental Material. (PDF)
