# Supplementary material for: Integration of the Integrated Emergency Surgical Officers (IESO) initiative within the health system: Perspectives of non-physician surgeons in Ethiopia’s Tigray region
Source: PLoS One. 2026 Sep 28;21(9):e0358569. doi: 10.1371/journal.pone.0358569 (PMC13618940; doi:10.1371/journal.pone.0358569)
Supplement: Supplementary file 1 [file pone.0358569.s001.pdf]

Supplementary Material

Integration of the Integrated Emergency Surgical Officers (IESO) Initiative within the Health System: Perspectives of Non-Physician Surgeons in Ethiopia’s Tigray Region

Manuscript ID: PONE-D-25-45486

This file compiles the quantitative and qualitative data-collection instruments and the pre-test used in the study.

Section	Contents
S1 File	Survey on the Training (KoboToolbox export)
S2 File	Quantitative Questionnaire: Survey on the Practice of Integrated Emergency Surgical Officers (IESOs) in the Tigray Region
S3 File	IESO Training-Related Questionnaire
S4 File	Pre-test for Safe Cesarean Delivery for IESOs
S5 File	Focus Group Discussion Guide for Integrated Emergency Surgical Officers (IESOs) in the Tigray Region
S6 File	In-depth Interview Guide for Integrated Emergency Surgical Officers (IESOs) in the Tigray Region

S1 File

Survey on the Training (KoboToolbox export)

Survey on the Training

Introduction and Consent: Hello. My name is _. I am working with the Safe Surge project from Mekelle University College of Health Sciences (MUCHS), and Tigray Regional Health Bureau (TRHB. We are now conducting a survey related to the TRAINING (CS, essential newborn care and obstetric anaesthesia). Such information would help to enhance the training for the future. I know you are busy, so this will take only 15 minutes. Your participation is completely voluntary and anonymous. All of the answers you give will be confidential and will not be shared with anyone other than members of our survey team. You don't have to be in the survey, but we hope you will agree to answer the questions since your answers to the question are important. If I ask any question you don't want to answer, just let me know and I will go on to the next question or you can stop the interview at any time. In addition, the data will be collected through an electronic data collection called Kobo Toolbox.

---

1. Cohort of IESO training

- ◯ Cohort I
- ◯ Cohort II

2. Gender/ Sex

- ◯ Male
- ◯ Female

3. Age

____

4. Name of the health facility

____

5. Type of the health facility

- ◯ Health center
- ◯ Primary hospital
- ◯ General hospital

6. What was your profession before joining an IESO

- ◯ HO
- ◯ Midwives
- ◯ BSc Nurse

Objectives, communication and participation in the training

7.In your opinion, was the training's objectives/aims related to your profession?

- Not at all
- Slightly
- Moderately
- Significantly
- Extremely

8. Level of active participation or two-way communication in the training

- Not at all
- Slightly
- Moderately
- Significantly
- Extremely

9. Level of having a smooth communication in the training process

- Not at all
- Slightly
- Moderately
- Significantly
- Extremely

11. The importance of having a pre and post-test in the training

- Not at all
- Slightly
- Moderately
- Significantly
- Extremely

III:- Level of understanding BEFORE taking of the training

---

12. How high was your level of understanding to the history of caesarean delivery before taking the training?

- Not at all
- Slightly
- Moderately High
- Significantly High
- Extremely High

13. How high was your level of understanding to the indication for caesarean delivery and safe prevention before taking the training?

- ◯ Not at all
- ◯ Slightly
- ◯ Moderately High
- ◯ Significantly High
- ◯ Extremely High

14. How high was your level of understanding to the indication of Pre-OP preparation before taking the training?

- ◯ Not at all
- ◯ Slightly
- ◯ Moderately High
- ◯ Significantly High
- ◯ Extremely High

15. How high was your level of understanding to the surgical techniques before taking the training?

- ◯ Not at all
- ◯ Slightly
- ◯ Moderately High
- ◯ Significantly High
- ◯ Extremely High

16. How high was your level of understanding to the difficulty caesarean delivery before taking the training?

- ◯ Not at all
- ◯ Slightly
- ◯ Moderately High
- ◯ Significantly High
- ◯ Extremely High

17. How high was your level of understanding to the early neonatal care before taking the training?

- ◯ Not at all
- ◯ Slightly
- ◯ Moderately High
- ◯ Significantly High
- ◯ Extremely High

18. How high was your level of understanding to the obstetrics anaesthesia before taking the training?

- ◯ Not at all
- ◯ Slightly
- ◯ Moderately High
- ◯ Significantly High
- ◯ Extremely High

IV:- Level of understanding after the provision of the training

19. How has this training improved your understanding of the history of Cesarean delivery?

- ◯ Not at all
- ◯ Slightly
- ◯ Moderately
- ◯ Significantly
- ◯ Extremely

20. How has this training improved your understanding of indications for Cesarean delivery?

- ◯ Not at all
- ◯ Slightly
- ◯ Moderately
- ◯ Significantly
- ◯ Extremely

21. How has this training improved your understanding of preoperative preparation for Cesarean delivery?

- ◯ Not at all
- ◯ Slightly
- ◯ Moderately
- ◯ Significantly
- ◯ Extremely

22. How has this training improved your understanding of surgical techniques of Cesarean delivery?

- ◯ Not at all
- ◯ Slightly
- ◯ Moderately
- ◯ Significantly
- ◯ Extremely

23. How has this training improved your understanding of performing difficult Cesarean delivery

- ◯ Not at all
- ◯ Slightly
- ◯ Moderately
- ◯ Significantly
- ◯ Extremely

24. How has this training improved your skill of performing neonatal resuscitation?

- ◯ Not at all
- ◯ Slightly
- ◯ Moderately
- ◯ Significantly
- ◯ Extremely

25. How has this training improved your understanding of obstetric anesthesia?

- ◯ Not at all
- ◯ Slightly
- ◯ Moderately
- ◯ Significantly
- ◯ Extremely

Overall perception about the training

---

26. Which section of the training was the most easiet to capture/understand (Check all that apply)

- ◯ History of caesarean delivery
- ◯ Caesarean delivery and safe prevention
- ◯ Pre-OP preparation
- ◯ Surgical techniques
- ◯ Difficulty caesarean delivery
- ◯ Early neonatal care
- ◯ Obstetrics anaesthesia

27. Which section of the training was the most difficult to capture/understand (Check all that apply)

- ◯ History of caesarean delivery
- ◯ Caesarean delivery and safe prevention
- ◯ Pre-OP preparation
- ◯ Surgical techniques
- ◯ Difficulty caesarean delivery
- ◯ Early neonatal care
- ◯ Obstetrics anaesthesia

28. Overall level of obtaining new knowledge from the training

- ◯ Not at all
- ◯ Slightly
- ◯ Moderately
- ◯ Significantly
- ◯ Extremely

29. Overall, the training content to update your level of knowledge

- ◯ Not at all
- ◯ Slightly
- ◯ Moderately
- ◯ Significantly
- ◯ Extremely

30. Overall satisfaction on the training content

- ◯ Not at all
- ◯ Slightly
- ◯ Moderately
- ◯ Significantly
- ◯ Extremely

31. Will this training change some of your clinical practice?

- ◯ Not at all
- ◯ Slightly
- ◯ Moderately
- ◯ Significantly
- ◯ Extremely

32. Do you recommend such training for other IESO staffs

- ◯ Not at all
- ◯ Slightly
- ◯ Moderately
- ◯ Significantly
- ◯ Extremely

32. Would you participate in such kind of training in the future if you are asked for?

- ◯ Not at all
- ◯ Slightly
- ◯ Moderately
- ◯ Significantly
- ◯ Extremely

33. Area of improvement of the training in the future [Check all that apply]

- [ ] Supported the training with practice/ clinical aspect
- [ ] Having a training on a surgery side
- [ ] Providing on job training
- [ ] Increase number of training days

Thank you !!!

S2 File

Quantitative Questionnaire: Survey on the Practice of Integrated Emergency Surgical Officers (IESOs) in the Tigray Region

Safe CS surgery

Quantitative Questionnaire Title: Survey on the Practice of Integrated Emergency Surgical Officers (IESOs) in the Tigray Region: Good morning/afternoon. My name is _. This survey is being conducted by TRHB and MUCHS. The aim of the survey is to assess the Practice of Integrated Emergency Surgical Officers (IESOs) in the Tigray Region. This survey provides us with the necessary information that will help to strengthen the IESO practice in the Tigray region. There is no risk to you if you participate in this survey. The information you will share with us if you participate in this survey will be kept completely confidential. The answers you give to any questions are completely anonymous since your names will not be written on the questionnaire. Participation in this survey is voluntary. If you agree to participate in this survey, you will be asked to answer some questions related to it. The interview will take 30 - 45 minutes. If you agree to record the interview, your responses will be documented. Your information and responses will be securely stored in a location only accessible by the research team and will be destroyed after this project as soon as possible. Please complete the following questionnaire. Your responses are confidential and will be used solely for research purposes.

Socio-demographic

1. Age

____

2. Gender (Sex)

- ◯ Male
- ◯ Female

2.1. Marital status

- ◯ Married
- ◯ Single
- ◯ Widowed
- ◯ Divoced

3. Years of Experience as an IESO

In years

____

3.1. Batch of IESO

- ◯ First batch
- ◯ Second batch
- ◯ Third batch
- ◯ Fourth batch

4. Institution Name

____

5. Woreda or District Name

____

6. Which type of services/infrastructure are availble currently in your facility ? (Check all that apply)

- [ ] Laboratory service
- [ ] Pharmacy
- [ ] X-ray machine
- [ ] Ambulance service
- [ ] Radiology service
- [ ] Sterilization facility
- [ ] Oxygen source
- [ ] Backup power supply
- [ ] Blood transfusion service
- [ ] Separate MCH unit
- [ ] Water supply
- [ ] Access to public transport
- [ ] Laundry service

7. Type of Facility:

- ○ Health Center
- ○ Primary Hospital
- ○ General Hospital
- ○ Other

8. Are you currently working as an IESO

- ○ Yes
- ○ No

9. When was the last time you work as an IESO

yyyy-mm-dd ____________________

____

Please specify

____

Training and competence

10. Rate the adequacy of your training for performing emergency obstetric procedures

- Very Low/Inadequate
- Low
- Neutral
- High
- Very High

11. Rate the overall competency of your training for performing emergency obstetric procedures

- Very Low/Inadequate
- Low
- Neutral
- High
- Very High

12. How much adequate to invests time and energy in self-development and growth.

- Very Low/Inadequate
- Low
- Neutral
- High
- Very High

13. How much adequate to remains open to new ideas and approaches

- Very Low/Inadequate
- Low
- Neutral
- High
- Very High

14. How much adequate to builds trust and open communication among team members

- Very Low/Inadequate
- Low
- Neutral
- High
- Very High

15. How much adequate to takes steps to resolve conflict situations

- ◯ Very Low/Inadequate
- ◯ Low
- ◯ Neutral
- ◯ High
- ◯ Very High

16. How much adequate to takes responsibility for actions

- ◯ Very Low/Inadequate
- ◯ Low
- ◯ Neutral
- ◯ High
- ◯ Very High

17. How much adequate to exercises good judgment by making sound and well-informed decisions

- ◯ Very Low/Inadequate
- ◯ Low
- ◯ Neutral
- ◯ High
- ◯ Very High

18. How much adequate to maintains a professional demeanor in stressful and difficult situations

- ◯ Very Low/Inadequate
- ◯ Low
- ◯ Neutral
- ◯ High
- ◯ Very High

19. How much adequte to uses qualitative and quantitative data and analytical tools in problem solving.

- ◯ Very Low/Inadequate
- ◯ Low
- ◯ Neutral
- ◯ High
- ◯ Very High

20. How much adequte to empowers others by sharing power and authority

- ◯ Very Low/Inadequate
- ◯ Low
- ◯ Neutral
- ◯ High
- ◯ Very High

21. How much adequte to persuades others to consider alternative points of view.

- ◯ Very Low/Inadequate
- ◯ Low
- ◯ Neutral
- ◯ High
- ◯ Very High

22. How confident are you in performing emergency surgical procedures independently?

- ◯ Not at all confident
- ◯ Slightly confident
- ◯ Moderately confident
- ◯ Very confident
- ◯ Extremely confident

23. How confident are you in reffering mothers with sever complication to higher level ?

- ◯ Not at all confident
- ◯ Slightly confident
- ◯ Moderately confident
- ◯ Very confident
- ◯ Extremely confident

24. How confident are you in reffering neonate with complication to higher level ?

- ◯ Not at all confident
- ◯ Slightly confident
- ◯ Moderately confident
- ◯ Very confident
- ◯ Extremely confident

25. How confident are you in writting feedback fo refferral cases ?

- ◯ Not at all confident
- ◯ Slightly confident
- ◯ Moderately confident
- ◯ Very confident
- ◯ Extremely confident

26. How confident are you in providing neonatal resuscitation ?

- ◯ Not at all confident
- ◯ Slightly confident
- ◯ Moderately confident
- ◯ Very confident
- ◯ Extremely confident

27. How confident are you in consulting staff/colleques about a certain issue/case ?

- ◯ Not at all confident
- ◯ Slightly confident
- ◯ Moderately confident
- ◯ Very confident
- ◯ Extremely confident

28. Have you ever received training on communication skill ?

- ◯ Yes
- ◯ No

29. Level of communication skill with your colleagues

- ◯ Very low
- ◯ Low
- ◯ Neutral
- ◯ High
- ◯ Very High

30. Level of communication skill with patients

- ◯ Very low
- ◯ Low
- ◯ Neutral
- ◯ High
- ◯ Very High

31. Level of communication skill with patients relatives

- ◯ Very low
- ◯ Low
- ◯ Neutral
- ◯ High
- ◯ Very High

32. Have you ever received training on research methods ?

- ◯ Yes
- ◯ No

33. Have you ever received training on quality improvement ?

- ◯ Yes
- ◯ No

34. Do you attend normal vaginal deliveries?

- ◯ Yes
- ◯ No

35. If yes, how frequently do you attend normal vaginal deliveries?

- ◯ Rarely
- ◯ Sometimes
- ◯ Often
- ◯ Very often

36. Do you perform operative vaginal deliveries (e.g., forceps or vacuum extraction)?

- ◯ Yes
- ◯ No

37. If yes, how frequently do you perform operative vaginal deliveries?

- ◯ Rarely
- ◯ Sometimes
- ◯ Often
- ◯ Very often

38. Do you perform Cesarean deliveries?

- ◯ Yes
- ◯ No

39. If yes, how frequently do you perform Cesarean deliveries?

- ◯ Rarely
- ◯ Sometimes
- ◯ Often
- ◯ Very often

40. How frequently have you performed B-lynch suture application?

- ◯ Never
- ◯ Rarely
- ◯ Sometimes
- ◯ Often
- ◯ Very often

41. How frequently have you performed uterine artery ligation?

- ◯ Never
- ◯ Rarely
- ◯ Sometimes
- ◯ Often
- ◯ Very often

42. How frequently have you performed peripartum hysterectomy?

- ◯ Never
- ◯ Rarely
- ◯ Sometimes
- ◯ Often
- ◯ Very often

43. Approximately how many surgeries do you perform per week?
In number

____

44. Approximately how many surgeries do you perform per month?
In number

____

45. Approximately how many surgeries do you perform per year?

____

46. Approximately how many Normal Vaginal Deliveries do you attend per month ?

____

47. Approximately how many Operative Vaginal Deliveries do you attend per month ?

____

48. Approximately how many Cesarean Deliveries do you attend per month ?

____

49. Approximately how many Emergency Gynecological Procedures do you attend per month ?

____

50. Approximately how many Other Emergency surgeries Procedures do you attend per month ?

____

51. Approximately how many total deliveries (vaginal + cesarean + operative) performed in your facility per year ? (annual figures on the preceding fiscal year)

____

52. Approximately how many cesarean section performed in your facility? (annual figures on the preceding fiscal year)

____

53. Number of Perintal mortality (annual figures on the preceding fiscal year) in your facility

____

54. Number of Perintal mortality (in the past 5 years) in your facility

____

55. Number of Maternal mortality (annual figures on the preceding fiscal year) in your facility

____

56. Number of Maternal mortality (in the past 5 year) in your facility

____

57. Is there anyone else trained to do surgery in your institution ?

- ◯ Yes
- ◯ No

58. If yes, who trained to do surgery in your instituion ?

- [ ] Another IESO
- [ ] General Surgeon
- [ ] Clinical Midwifery
- [ ] Obstetrician and gynecologist

59. Is there a health professional who is trained in neonatal resuscitation in your facility?

- ◯ Yes
- ◯ No

60. If yes what is his/her profession?

- ◯ IESO
- ◯ Midwifery
- ◯ NICU nurse
- ◯ General practitioner
- ◯ Pediatrician
- ◯ Gynecologist

61. How many staff trained about neontal resustation?

____

62. Overall, how satisfied are you with your role as an IESO?

- ◯ Very Dissatisfied
- ◯ Dissatisfied
- ◯ Neutral
- ◯ Satisfied
- ◯ Very Satisfied

62.1. Overall, how satisfied ae you with your role as an IESO ? Write you answer that ranges from 0 to 10 [0=Lowest and 10=Highest]

____

63. Rate the support you receive from hospital administration /wereda health office ?

- ◯ Very Poor
- ◯ Poor
- ◯ Neutral
- ◯ Good
- ◯ Very Good

64. Rate the collaboration with OR team:

- ◯ Very Poor
- ◯ Poor
- ◯ Neutral
- ◯ Good
- ◯ Very Good

65. Rate the collaboration with other health care professionals (Other than OR team):

- ◯ Very Poor
- ◯ Poor
- ◯ Neutral
- ◯ Good
- ◯ Very Good

66. Rate the collaboration with managment team:

- ◯ Very Poor
- ◯ Poor
- ◯ Neutral
- ◯ Good
- ◯ Very Good

67. Do you feel your work is adequately recognized by the managment body/higher official ?

- ◯ Strongly Disagree
- ◯ Disagree
- ◯ Neutral
- ◯ Agree
- ◯ Strongly Agree

68. Do you feel your work is adequately recognized by the community ?

- ◯ Strongly Disagree
- ◯ Disagree
- ◯ Neutral
- ◯ Agree
- ◯ Strongly Agree

69. Do you feel your work is adequately appreciated by the managment body/higher official?

- Strongly Disagree
- Disagree
- Neutral
- Agree

70. Do you feel your work is adequately appreciated by the community?

- Strongly Disagree
- Disagree
- Neutral
- Agree

71. To what extent do you need additional training ?

- Not at all
- Slightly
- Moderately
- Very much
- Extremely

72. To what extent do you need continuing education ?

- Not at all
- Slightly
- Moderately
- Very much

73. Select the resources you lack in your practice (check all that apply):

- [ ] Surgical Equipment
- [ ] Medications
- [ ] Adequte staffing
- [ ] Continuing Education Opportunities
- [ ] Support from Specialists
- [ ] Others

If other (please specify)

____

74. Main challenges faced in daily practice (check all that apply):

- [ ] High Patient Load
- [ ] Limited Resources
- [ ] Lack of Training
- [ ] Administrative Issues
- [ ] Communication with Staff
- [ ] Others (please specify):

Please specify

____

75. Do you believe that the IESO program/profession improves healthcare access in rural areas?

- ○ Strongly Disagree
- ○ Disagree
- ○ Neutral
- ○ Agree
- ○ Strongly Agree

76. Do you believe that the IESO program/profession contributed in reduction of maternal mortality in the region ?

- ○ Strongly Disagree
- ○ Disagree
- ○ Neutral
- ○ Agree
- ○ Strongly Agree

77. Do you believe that the IESO program contributed in reduction of perinatal/neonatal mortality in the region ?

- ○ Strongly Disagree
- ○ Disagree
- ○ Neutral
- ○ Agree
- ○ Strongly Agree

78. Would you recommend the IESO profession to others?

- ○ Definitely Not
- ○ Probably Not
- ○ Not Sure
- ○ Probably Yes
- ○ Definitely Yes

79. Likelihood of continuing as an IESO in the next three years:

- Very Unlikely
- Unlikely
- Neutral
- Likely
- Very likey

80. Likelihood of continuing as an IESO in the next five years:

- Very Unlikely
- Unlikely
- Neutral
- Likely
- Very likey

81. Likelihood of continuing as an IESO in the next ten years:

- Very Unlikely
- Unlikely
- Neutral
- Likely
- Very likey

82. Do you have an intention to shift your profession in the coming 3 years ?

- Yes
- No
- Neutral

83. For what profession are you intend to shift in the coming 3 years?

- Medicine
- Public health

84. Acceptance of your profession by Obstetric and Gynaecologist specialist

- Very Low Accepted
- Low Accepted
- Moderate
- Higly Accepted
- Very High Accepted

85. Acceptance of your profession by surgeon

- ◯ Very Low Accepted
- ◯ Low Accepted
- ◯ Moderate
- ◯ Higly Accepted
- ◯ Very High Accepted

86. Conflict of interest with Obstetric and Gynaecologist specialist

- ◯ Very Low Accepted
- ◯ Low Accepted
- ◯ Moderate
- ◯ Higly Accepted
- ◯ Very High Accepted

87. Conflict of interest with surgeons

- ◯ Very Low Accepted
- ◯ Low Accepted
- ◯ Moderate
- ◯ Higly Accepted
- ◯ Very High Accepted

Thank you for your precious time !!!

S3 File

IESO Training-Related Questionnaire

<u>IESO Training Related Questionnaire</u>

Introduction and Consent:

Hello. My name is ____. I am working with the Safe Surge project from Mekelle University College of Health Sciences (MUCHS), and Tigray Regional Health Bureau (TRHB. We are now conducting a survey related to the TRAINING (CS, essential newborn care and obstetric fistula). Such information would help to enhance the training for the future. I know you are busy, so this will take only a few minutes approximately 15 minutes. Your participation is completely voluntary and anonymous. All of the answers you give will be confidential and will not be shared with anyone other than members of our survey team. You don't have to be in the survey, but we hope you will agree to answer the questions since your answers to the question are important. If I ask any question you don't want to answer, just let me know and I will go on to the next question or you can stop the interview at any time. In addition, the data will be collected through an electronic data collection called Kobo Toolbox.

Are you willing to participate in the Survey related to IESO TRAINING

- Yes
- No

I:- **Sociodemographic Characteristics**

1. Cohort of IESO training
    a. Cohort I
    b. Cohort II
2. Gender/ Sex
    a. Male
    b. Female
3. Age:____
4. Years of experience as an IESO:_________years
5. Name of the health facility: _____
6. Type of the health facility
    a. Health center
    b. Primary hospital
    c. General hospital
7. What was your profession before joining an IESO
    - HO
    - Midwives
    - BSc Nurse

II:- **Objectives, communication and participation in the training**

8. How do you see the relatedness of the training aim/objective with your professional background
    - Not at all
    - Slightly
    - Moderately
    - Significantly
    - Extremely
9. Level of active participation or two-way communication in the training
    a. Not at all
    b. Slightly
    c. Moderately
    d. Significantly
    e. Extremely
10. Level of having a smooth communication in the training process
    a. Not at all
    b. Slightly
    c. Moderately
    d. Significantly
    e. Extremely
11. The importance of having a pre and post-test in the training
    a. Not at all
    b. Slightly
    c. Moderately
    d. Significantly
    e. Extremely

III:- Level of understanding before the taking of the training

12. How was your level of understanding to the history of caesarean delivery before the taking training?
    a. Not at all
    b. Slightly
    c. Moderately
    d. Significantly
    e. Extremely
13. How was your level of understanding to the indication for caesarean delivery and safe prevention before the taking training?
    a. Not at all
    b. Slightly
    c. Moderately
    d. Significantly
    e. Extremely
14. How was your level of understanding to the indication of Pre-OP preparation before taking the training?

    a. Not at all
    b. Slightly

   c. Moderately
   d. Significantly
   e. Extremely
15. How was your level of understanding to the surgical techniques before taking the training?
   a. Not at all
   b. Slightly
   c. Moderately
   d. Significantly
   e. Extremely
16. How was your level of understanding to the difficulty caesarean delivery before taking the training?
   a. Not at all
   b. Slightly
   c. Moderately
   d. Significantly
   e. Extremely
17. How was your level of understanding to the early neonatal care before taking the training?
   a. Not at all
   b. Slightly
   c. Moderately
   d. Significantly
   e. Extremely

18. How was your level of understanding to the obstetrics anaesthesia before taking the training?
   a. Not at all
   b. Slightly
   c. Moderately
   d. Significantly
   e. Extremely

IV:- Level of understanding after the provision of the training

19. How well do understand the history of caesarean delivery now that you've completed the training?
   a. Not at all
   b. Slightly
   c. Moderately
   d. Significantly
   e. Extremely
20. How well do understand the indication for caesarean delivery and safe prevention now that you've completed the training?

   a. Not at all
   b. Slightly
   c. Moderately

    d. Significantly
    e. Extremely
21. How well do you understand the indication for Pre-OP preparation now that you've completed the training?
    a. Not at all
    b. Slightly
    c. Moderately
    d. Significantly
    e. Extremely
22. How well do you understand the surgical techniques delivery now that you've completed the training?
    a. Not at all
    b. Slightly
    c. Moderately
    d. Significantly
    e. Extremely
23. How well do you understand the difficulty caesarean delivery now that you've completed the training?
    a. Not at all
    b. Slightly
    c. Moderately
    d. Significantly
    e. Extremely
24. How well do you understand early neonatal care now that you've completed the training?
    a. Not at all
    b. Slightly
    c. Moderately
    d. Significantly
    e. Extremely
25. How well do you understand obstetric anaesthesia now that you've completed the training?
    a. Not at all
    b. Slightly
    c. Moderately
    d. Significantly
    e. Extremely

IV: Overall perception about the training

26. Which section of the training was the easiest to capture/understand (**Check all that apply**)
    - History of caesarean delivery
    - Caesarean delivery and safe prevention

- Pre-OP preparation
- Surgical techniques
- Difficulty caesarean delivery
- Early neonatal care
- Obstetrics anaesthesia

27. Which section of the training was the most difficult to capture/understand (**Check all that apply**)
    - History of caesarean delivery
    - Caesarean delivery and safe prevention
    - Pre-OP preparation
    - Surgical techniques
    - Difficulty caesarean delivery
    - Early neonatal care
    - Obstetrics anaesthesia
28. Level of obtaining new knowledge from the training
    a. Not at all
    b. Slightly
    c. Moderately
    d. Significantly
    e. Extremely
29. Overall, the training content to update your level of knowledge
    a. Not at all
    b. Slightly
    c. Moderately
    d. Significantly
    e. Extremely
30. How satisfied were you with the overall execution of the training
    a. Not at all
    b. Slightly
    c. Moderately
    d. Significantly
    e. Extremely
31. Do you recommend such training for other IESO staffs
    a. Not at all
    b. Slightly
    c. Moderately
    d. Significantly
    e. Extremely
32. Would you participate in such kind of training in the future if you are asked for?
    a. Not at all
    b. Slightly
    c. Moderately
    d. Significantly
    e. Extremely
33. Area of improvement of the training in the future [**Check all that apply**]
    - Supported the training with practice/ clinical aspect

- Having a training on a surgery side
- Providing on job training
- Increase number of training days

S4 File

Pre-test for Safe Cesarean Delivery for IESOs

November 2024

Pre-test for Safe Cesarean Delivery for IESOs

Instruction: Choose Single Best Answer (2 Points Each)

1. A 35-year-old primigravida at 39 weeks gestation has a history of a prior myomectomy that involved entry into the uterine cavity. She desires a vaginal delivery. What is the most appropriate management?
    a. Allow trial of labor with close monitoring
    b. Schedule an elective Cesarean delivery at 39 weeks
    c. Induce labor with oxytocin at 40 weeks
    d. Recommend immediate Cesarean hysterectomy
2. During a scheduled Cesarean delivery, placenta accreta involving the anterior uterine wall is encountered. What is the most appropriate intraoperative management?
    a. Manual removal of the placenta and repair of the uterine defect
    b. Leave the placenta in situ and close the uterus
    c. Perform a Cesarean hysterectomy
    d. Administer methotrexate postoperatively
3. A patient undergoing emergency Cesarean delivery under spinal anesthesia develops severe hypotension and bradycardia intraoperatively. What is the most likely cause?
    a. High spinal block affecting cardiac sympathetic fibers
    b. Anaphylactic reaction to anesthetic agents
    c. Vasovagal reaction to surgical manipulation
    d. Amniotic fluid embolism
4. A 25-year-old woman scheduled for elective Cesarean delivery has a platelet count of 70,000/μL. Which anesthetic technique is most appropriate?
    a. General anesthesia
    b. Spinal anesthesia
    c. Epidural anesthesia
    d. Local anesthesia with sedation

5. A 35-year-old G2P1 woman with a history of a previous classical Cesarean section presents at 37 weeks gestation in active labor. She was not scheduled for a repeat Cesarean delivery and is now 8 cm dilated upon admission. Fetal monitoring shows a reassuring heart rate. What is the most appropriate management at this point?
    a. Allow labor to continue with close monitoring for uterine rupture
    b. Prepare for immediate repeat Cesarean delivery
    c. Administer tocolytics to stop labor and schedule Cesarean delivery
    d. Proceed with assisted vaginal delivery to expedite labor

6. During a Cesarean delivery, after delivery of the infant and placenta, the patient experiences sudden, severe hypotension, and the surgical field fills with blood. Inspection reveals a large broad ligament hematoma expanding rapidly. What is the most appropriate next step in management?
    a. Administer uterotonics to control bleeding
    b. Perform uterine artery embolization
    c. Surgically explore the broad ligament and ligate the bleeding vessels
    d. Close the abdomen and transfer to interventional radiology
7. Which of the following intraoperative complications is a patient with preeclampsia most at risk for during Cesarean delivery?
    a. Placental abruption
    b. Pulmonary edema
    c. Uterine atony
    d. Disseminated intravascular coagulation (DIC)
8. What is the recommended antibiotic prophylaxis for Cesarean delivery to reduce the risk of postoperative infection?
    a. Single dose of intravenous ceftriaxone before incision
    b. Single dose of intravenous cefazolin before incision
    c. Oral doxycycline started postoperatively
    d. Intravenous ampicilline during surgery
9. Prior Cesarean delivery increases the risk of which placental complication in subsequent pregnancies?
    a. Placenta previa
    b. Placental abruption
    c. Placenta accreta
    d. Both A and C
10. During Cesarean delivery, accidental injury to the uterine artery occurs. What is the immediate next step?
    a. Apply direct pressure to control bleeding
    b. Clamp and ligate the bleeding vessel
    c. Proceed with hysterectomy
    d. Call for vascular surgery consultation
11. A pregnant woman with HIV and a viral load of 1,200 copies/mL at 38 weeks gestation is scheduled for delivery. What is the best method to reduce vertical transmission?
    a. Scheduled Cesarean delivery before labor onset
    b. Allow vaginal delivery with antiretroviral therapy
    c. Administer intrapartum zidovudine and proceed with Cesarean delivery
    d. Delay delivery until viral load is undetectable
12. In a patient with placenta previa scheduled for Cesarean delivery, which blood products should be immediately available?
    a. Packed red blood cells only
    b. Fresh frozen plasma and platelets

    c. Massive transfusion protocol products
    d. Autologous blood donation
13. A patient with placenta percreta requires Cesarean hysterectomy. What is the most significant intraoperative risk?
    a. Bladder injury
    b. Massive hemorrhage
    c. Infection
    d. Thromboembolism
14. For a preterm fetus at 28 weeks gestation requiring Cesarean delivery, which uterine incision is preferred?
    a. Low transverse incision
    b. Low vertical incision
    c. Classical vertical incision
    d. Inverted T incision
15. A patient experiences nausea and vomiting during Cesarean delivery under spinal anesthesia. What is the most likely cause?
    a. Hypotension due to sympathetic blockade
    b. Reaction to opioids in the spinal anesthesia
    c. Uterine massage
    d. Adverse effect of antibiotics
16. Which intraoperative practice reduces the formation of postoperative adhesions after Cesarean delivery?
    a. Peritoneal closure
    b. Leaving the peritoneum open
    c. Liberal use of adhesion barriers
    d. Minimizing tissue handling and desiccation
17. A woman desires tubal sterilization during her scheduled Cesarean delivery. Which is true regarding consent?
    a. Consent can be obtained at any time before the procedure
    b. Sterilization consent requires a waiting period in some jurisdictions
    c. Consent is implied with the Cesarean delivery consent
    d. No additional consent is needed if the patient is over 35
18. Which method of skin closure in Cesarean delivery is associated with reduced wound complications in obese women?
    a. Staples
    b. Subcuticular sutures
    c. Interrupted nylon sutures
    d. Delayed primary closure
19. A postpartum woman develops a deep vein thrombosis after Cesarean delivery. Which anticoagulant is safe during breastfeeding?
    a. Warfarin

b. Low molecular weight heparin
c. Direct oral anticoagulants (DOACs)
d. Aspirin

20. What is an advantage of the Joel-Cohen incision over the Pfannenstiel incision?
    a. Shorter operative time
    b. Less postoperative pain
    c. Improved cosmetic result
    d. Lower risk of incisional hernia
21. During Cesarean delivery, thick meconium-stained amniotic fluid is noted. What is the immediate management for the neonate?
    a. Routine neonatal care without intervention
    b. Immediate intubation and tracheal suctioning
    c. Begin chest compressions
    d. Administer prophylactic antibiotics
22. Which intraoperative intervention reduces the risk of postpartum hemorrhage during Cesarean delivery?
    a. Uterine massage before placental delivery
    b. Prophylactic administration of oxytocin after delivery
    c. Routine use of uterine artery ligation
    d. Delayed cord clamping
23. A low-lying placenta is identified at 32 weeks gestation. What is the most appropriate management?
    a. Schedule Cesarean delivery at 37 weeks
    b. Repeat ultrasound at 36 weeks
    c. Allow trial of labor
    d. Administer steroids and monitor
24. Shortly after delivery of the fetus during Cesarean section, the patient becomes hypotensive and hypoxic. Which diagnosis should be immediately considered?
    a. Amniotic fluid embolism
    b. Anesthetic overdose
    c. Myocardial infarction
    d. Pulmonary edema
25. In pregestational diabetic patients undergoing Cesarean delivery, which preoperative management is crucial?
    a. Discontinue insulin on the day of surgery
    b. Maintain euglycemia with insulin infusion
    c. Administer steroids for fetal lung maturity
    d. Schedule surgery in the late afternoon

26. A large lower uterine segment fibroid obstructs access during Cesarean delivery. What is the appropriate management?
    a. Perform myomectomy to remove the fibroid
    b. Extend the uterine incision around the fibroid
    c. Convert to a classical Cesarean incision
    d. Abandon the procedure and plan for hysterectomy
27. Delayed cord clamping during Cesarean delivery is associated with:
    a. Increased neonatal iron stores
    b. Reduced maternal blood loss
    c. Decreased risk of neonatal jaundice
    d. Lower incidence of postpartum hemorrhage
28. Which surgical technique during Cesarean delivery may reduce postoperative pain?
    a. Blunt dissection of tissues
    b. Closure of both visceral and parietal peritoneum
    c. Use of non-absorbable sutures
    d. Single-layer uterine closure
29. In women with hepatitis C infection, which statement is true regarding Cesarean delivery?
    a. Cesarean delivery reduces vertical transmission
    b. Breastfeeding is contraindicated
    c. No intervention has been proven to reduce transmission
    d. Antiviral therapy should be initiated immediately postpartum
30. Which factor increases the risk of uterine atony during Cesarean delivery?
    a. Administration of magnesium sulfate
    b. Prolonged use of oxytocin during labor
    c. Chorioamnionitis
    d. All of the above
31. During Cesarean delivery for placenta previa, the placenta overlies the uterine incision site. What is the best approach to deliver the fetus?
    a. Incise directly through the placenta
    b. Extend the incision upward to avoid the placenta
    c. Deliver the fetus from a high transverse incision
    d. Perform a classical vertical incision
32. Regarding antibiotic prophylaxis in Cesarean delivery, which is correct?
    a. Antibiotics should be given after cord clamping
    b. A single preoperative dose is sufficient in most cases
    c. Antibiotics should be continued for 24 hours postoperatively
    d. Prophylactic antibiotics are unnecessary if membranes are intact
33. Which is an absolute contraindication to regional anesthesia for Cesarean delivery?

a. Patient refusal
b. Mild coagulopathy
c. Preexisting neurological disease
d. Obesity

34. To reduce postoperative morbidity, WHO recommends antibiotic prophylaxis be given within 30-60minutes prior to skin incision. Which of the following is true?
    A. Repeat dose of cefazolin is recommended if the mother has more than 2previous cesarean section.
    B. Generally it is recommended to be given antibiotics 30min before procedure and 1hr after the cesarean section
    C. According to WHO it is recommended single dose ceftriaxone to be given 30min before skin incision is the standard.
    D. Two dose of cefazolin is recommended if the duration of surgery is prolonged more than 3hrs.
35. Which of the following is TRUE regarding prevention and management of PPH?
    A. Evidence for medical management of PPH at CS tends to be of higher quality than that for surgical management.
    B. Manual removal of placenta has lower risk of bleeding than continuous delivery of placenta.
    C. Regarding B-Lynch use it is only used for those that doesn't require fertility preservation
    D. The first step is uterine artery ligation if no responses use B-Lynch.
    E. All
36. Why is oxytocin administered by the Anesthetist?
    A. Helps to contract uterus to deliver the placenta
    B. Reduces risk of infection
    C. Detaches the placenta from the uterine wall
    D. Stabilizes the patients' blood pressure
37. The visceral peritoneum should be sutured
    A. True
    B. False
38. For two-layer suturing, why is it important the first layer of suturing is well buried by the second layer?
    a. Reduce the incidence of endometriosis
    b. Reduce blood loss
    c. Reduces postoperative pain
    d. Reduce risk of infection
39. Why may the surgeon anticipate great bleeding at the uterine angles?
    a. The tissues converge here
    b. Difficult technique used in this area
    c. They are difficult to suture
    d. Proximity of the uterine vessels
40. Why is it suggested to clean the paracolic gutters?
    a. Prevents formation of adhesions
    b. To clear the surgeons view

c. Blood left in the abdominal can lead to infection
d. If left it can breakdown sutures prematurely

41. What is the name of the deepest layer of muscle in the anterior abdominal wall?
    a. Illiacus
    b. Internal oblique
    c. Transverse abdominis
    d. External oblique
42. What benefit does Pfannenstiel incision offer over a midline incision?
    a. Improved cosmetic result
    b. Less postoperative pain
    c. Decreased rates of incisional hernia
    d. All of the above
43. Spontaneous delivery of the placenta with fundal massage compared with manual extraction has been shown to reduce the risk of what complication?
    a. Deep venous thrombosis
    b. Amniotic fluid embolism
    c. Postpartum infection
    d. Retained placenta
44. Subcutaneous tissue greater than what depth should be closed with suture to avoid wound disruption?
    A. 2 cm
    B. 4cm
    C. 6 cm
    D. 1 0 cm
45. You are performing a primary low-transverse cesarean delivery on a term patient for failure to progress. NOT True about the operative technique
    a. Joel-Cohen incision is associated with shorter operating time, less use of suture, less blood loss, less post-operative pain, less wound complications
    b. Pfanesstein incision is associated with shorter operating time, less use of suture, less blood loss, less post-operative pain, less wound complications
    c. Vaginal cleansing for women with ruptured membranes and/or in labor with Povidone-iodine solution before cesarean section decreases endomyometritis.
    d. During skin preparation Chlorhexidine-alcohol (alcohol-based) is superior to iodine-based solutions.
46. **What is considered a high-risk newborn?**

    A. Newborns that are born preterm or post-term.

    B. Newborn with jaundice in the first 24 hours.

    C. Newborns who were sick but have now recovered.

    D. All of the above.

47. **During the initial stages of positive-pressure (bag and mask) ventilation, you should breathe for the baby at a rate of ______ times per minute.**

A. 10 to 20

B. 20 to 40

C. 30 to 40

D. 40 to 60

48. **Where should you position yourself to use the resuscitation bag most effectively?**

A. At the head or foot of the newborn

B. At the side or head of the newborn

C. At the side or foot of the newborn

D. Anywhere but close to the baby

49. **At 90 minutes after birth, an 1800-gram baby is placed skin-to-skin with the mother and has a temperature of 36.7˚C. What should you do to help maintain the baby's temperature?**

A. Bathe the baby in warm water.

B. Place the baby in direct sunlight.

C. Assist the mother with continuous skin-to-skin care.

D. Place the baby on an open warmer set for high heat output.

50. **Which of the following is NOT a component of Essential Newborn Care?**

A. Skin-to-skin contact

B. Vitamin K administration

C. Chlorohexidine administration

D. Labelling

E. None

S5 File

Focus Group Discussion Guide for Integrated Emergency Surgical Officers (IESOs) in the Tigray Region

Qualitative Questionnaire
Title: ***Focused group discussion Guide for Integrated Emergency Surgical Officers (IESOs) in the Tigray Region***

Topic: - Sociodemographic characteristics of the study participants

Code	Batch<br>Batch 1=1<br>Batch 2=2<br>Batch 3=3<br>Batch 4=4	Age	Sex<br>1= Male<br>2=Female	Marital status<br>1= Married<br>2= widowed<br>3=Divorced<br>4=Single	Year of experience	Residence<br>1= Urban<br>2= Rural	Background<br>1: Health officer<br>2: Midwife<br>3: Nurse
**Code 1**							
**Code 2**							
**Code 3**							
**Code 4**							
**Code 5**							
**Code 6**							
**Code 7**							
**Code 8**							
**Code 9**							
**Code 10**							
**Code 11**							
**Code 12**							

Your participation in this study implies that you have automatically consenting to be involved in the study. We will be recording your voices; however, no personal identification will be appear either in the transcription or in the report of the project.

Instructions: Please reflect on the following questions and provide detailed responses based on your experiences.

1. **Describe your overall experience working as an IESO in the region? Before the war? During the war? After cessation of hostilities?**
    - *Service years?*
2. **What motivated you to become an IESO, and how has your journey been since you started?**
    - *How was the adequacy of the IESO training?*
    - *How do you perceive the competency of the IESO training?*
        - *Knowledge, skill and attitude*
        - *Communication, Conflict management, persuade others, problem solving, influence other*
    - *Do you Identify any gaps or limitations in the IESO program that you have observed.*
        - *How do you think these could be addressed?*
    - *What are your needs in terms of resources, training, or support to perform your duties more effectively?*
    - *Looking ahead, what are your aspirations within the IESO program, and what changes would you like to see implemented?*
        - *Do you recommend other staffs or profession to join the program?*
3. **Can you share specific instances/ experience where you felt your work made a significant impact on your daily activities?**
    - Can you describe your experiences attending/performing?
        - *Normal vaginal deliveries, Operative vaginal deliveries,*
        - *Caesarean deliveries?*
        - *B-Lynch suture, Peripartum hysterectomy, Uterine artery ligation*
    - Volume of the clients in your facilities / life experiences?
    - How does the volume of surgeries you perform affect your professional life and patient outcomes?
    - Impact of your work to;
        - *Reduce the burden of referral hospitals*
        - *Patients' satisfaction*
        - *Reduction of maternal and perinatal morbidity and mortality*

4. How confident are you in managing complications that can arise during caesarean delivery?

- *What are your observations regarding annual maternal and neonatal mortality rates in your hospital? What was the experience?*
    - *How do you feel your role influences these outcomes?*

5. **Discuss any opportunities that have arisen from your practice that have contributed to your professional growth?**

- *Recognition and appreciation you receive for your work from*
    - *the community, Staffs, higher official*
- *How do you perceive the support from hospital administration/Woreda health office and your colleagues to perform your routine activities?*

6. **What are the most significant challenges you face in your role? How do you cope with them? How should the government/ the health system help you mitigate the challenges?**

- *COVID 19, War*
- *Future carrier and motivation*
- *Conflict of interest and acceptance of your profession with OBY-GYN and surgeons*

7. Is there anything else you would like to add about your experience as an IESO that we haven't covered?

Thank you for sharing your valuable insights!

S6 File

In-depth Interview Guide for Integrated Emergency Surgical Officers (IESOs) in the Tigray Region

Qualitative Questionnaire

Title: ***IDI Guide for Integrated Emergency Surgical Officers (IESOs) in the Tigray Region***

Topic: - Sociodemographic characteristics of the study participants

- *Batch: ______*
- *Age: ______*
- *Sex: _____*
- *Marital status: _______*
- *Year of experience as an IESO: ___________*
- *Residence: ________*
- *Background*

Your participation in this study implies that you have automatically consenting to be involved in the study. We will be recording your voices; however, no personal identification will be appeared either in the transcription or in the report of the project.

Instructions: Please reflect on the following questions and provide detailed responses based on your experiences.

1. **How and why, they became health workers?**
   - Overall academic status/ achievement in the primary and secondary school?
   - Did you join health worker based on your interest?
     - Who influence to join the profession?
     - Any role model person to join the profession?
2. **What motivated you to become an IESO, and how has your journey been since you started?**
   - *How was the adequacy and competency (KAP) of the IESO training?*
   - *Do you identify any gaps or limitations in the IESO program that you have observed.*
   - *What was your first experience? How was that?*
     - *What was the case [OBY – GYN or Surgery]*
     - *What was the outcome?*
     - *How was your feeling at that time?*
     - *Reaction from the*
       - *Patients and their relatives,*
       - *Health facilities and colloquies*
     - *How do you describe your contribution to.*
       - *Reduce morbidity and mortality*
       - *Reduce burden/volume at referral health facilities*
       - *Patient satisfaction*
   - *What is your career aspiration for the future?*
   - How do you evaluate your current level of knowledge and skill status with the trend of advancing medical technology
   - *Do you recommend other staffs or profession to join the program?*

3. **How confident are you in managing complications that can arise during caesarean delivery/ surgeries?**
   - *Best experience you encounter in your lifetime experience as an IESO?*
   - *Bad experience you encounter in your lifetime experience as an IESO?*

4. **Challenges they face in their job and how they cope with them.**
   - COVID- 19,
   - War
     - **Describe your overall experience working as an IESO in the region?**
       - **Before the war?**
       - **During the war?**
       - **After cessation of hostilities?**
   - Conflict of interest and acceptance from *OBY-GYN and surgeons*
5. **Is there anything else you would like to add about your experience as an IESO that we haven't covered?**

Thank you for sharing your valuable insights!
